# Supplementary material for: Tooth wear as an indicator of acculturation process in remote Amazonian populations
Source: PLoS One. 2020 Apr 21;15(4):e0230809. doi: 10.1371/journal.pone.0230809 (PMC7173625; doi:10.1371/journal.pone.0230809)
Supplement: S1 File — (DOCX) [file pone.0230809.s001.docx]

| Table containing the age and the mean of tooth wear of each individual. | | |
| --- | --- | --- |
| Subject | Age | Mean of tooth wear |
| #1 | 13.17 | 0.05 |
| #2 | 13.83 | 0.30 |
| #3 | 13.25 | 0.35 |
| #4 | 14.92 | 0.45 |
| #5 | 14.83 | 0.50 |
| #6 | 14.50 | 0.10 |
| #7 | 14.17 | 0.25 |
| #8 | 14.42 | 0.70 |
| #9 | 15.42 | 0.05 |
| #10 | 15.17 | 0.30 |
| #11 | 15.67 | 0.25 |
| #12 | 15.92 | 0.45 |
| #13 | 15.08 | 0.45 |
| #14 | 15.42 | 0.65 |
| #15 | 15.75 | 0.50 |
| #16 | 15.17 | 0.20 |
| #17 | 15.58 | 0.50 |
| #18 | 16.92 | 0.60 |
| #19 | 16.50 | 0.45 |
| #20 | 16.25 | 0.00 |
| #21 | 16.58 | 0.05 |
| #22 | 16.33 | 0.30 |
| #23 | 16.33 | 0.15 |
| #24 | 16.42 | 0.40 |
| #25 | 16.42 | 0.15 |
| #26 | 17.50 | 0.40 |
| #27 | 17.17 | 0.90 |
| #28 | 17.25 | 0.55 |
| #29 | 17.92 | 0.25 |
| #30 | 17.67 | 0.40 |
| #31 | 17.58 | 0.15 |
| #32 | 17.42 | 0.15 |
| #33 | 17.17 | 0.55 |
| #34 | 17.17 | 0.40 |
| #35 | 17.67 | 0.00 |
| #36 | 18.58 | 0.28 |
| #37 | 18.25 | 0.75 |
| #38 | 18.08 | 0.20 |
| #39 | 18.00 | 0.20 |
| #40 | 18.00 | 0.50 |
| #41 | 18.25 | 0.70 |
| #42 | 18.25 | 0.35 |
| #43 | 18.92 | 0.65 |
| #44 | 19.42 | 0.30 |
| #45 | 19.25 | 0.75 |
| #46 | 19.08 | 1.10 |
| #47 | 19.92 | 0.70 |
| #48 | 19.25 | 0.45 |
| #49 | 19.25 | 0.45 |
| #50 | 20.08 | 0.55 |
| #51 | 20.33 | 0.60 |
| #52 | 20.92 | 0.20 |
| #53 | 20.08 | 0.20 |
| #54 | 20.83 | 0.70 |
| #55 | 20.75 | 0.40 |
| #56 | 21.17 | 0.56 |
| #57 | 21.83 | 0.55 |
| #58 | 21.17 | 0.65 |
| #59 | 22.25 | 1.45 |
| #60 | 22.25 | 0.55 |
| #61 | 23.58 | 0.45 |
| #62 | 25.00 | 0.45 |
| #63 | 25.25 | 0.50 |
| #64 | 27.17 | 0.70 |
| #65 | 27.50 | 0.75 |
| #66 | 28.92 | 0.25 |
| #67 | 29.67 | 0.60 |
| #68 | 29.83 | 1.75 |
| #69 | 29.83 | 0.85 |
| #70 | 30.00 | 0.50 |
| #71 | 30.33 | 0.65 |
| #72 | 30.83 | 1.05 |
| #73 | 31.33 | 0.50 |
| #74 | 32.75 | 1.20 |
| #75 | 33.83 | 0.45 |
| #76 | 33.08 | 1.80 |
| #77 | 34.50 | 0.40 |
| #78 | 35.17 | 1.25 |
| #79 | 35.08 | 0.75 |
| #80 | 35.92 | 0.83 |
| #81 | 35.92 | 0.90 |
| #82 | 35.33 | 0.20 |
| #83 | 37.50 | 0.45 |
| #84 | 37.25 | 1.05 |
| #85 | 38.75 | 1.00 |
| #86 | 41.42 | 0.75 |
| #87 | 44.17 | 1.25 |
| #88 | 46.75 | 1.05 |
| #89 | 47.92 | 2.30 |
| #90 | 53.83 | 2.33 |
| #91 | 54.92 | 2.60 |
| #92 | 59.08 | 1.89 |
| #93 | 60.67 | 2.35 |
| #94 | 61.92 | 0.85 |
